# Phytotoxic, insecticidal, and antimicrobial activities of *Ajania tibetica* essential oil

**DOI:** 10.3389/fpls.2022.1028252

**Published:** 2022-11-18

**Authors:** Caixia Han, Shixing Zhou, Yu Mei, Qiumei Cao, Kai Shi, Hua Shao

**Affiliations:** ^1^ State Key Laboratory of Desert and Oasis Ecology, Xinjiang Institute of Ecology and Geography, Chinese Academy of Sciences, Urumqi, China; ^2^ University of Chinese Academy of Sciences, Beijing, China; ^3^ Research Center for Ecology and Environment of Central Asia, Xinjiang Institute of Ecology and Geography, Chinese Academy of Sciences, Urumqi, China

**Keywords:** *Ajania tibetica*, essential oil, phytotoxicity, insecticidal activity, antimicrobial activity

## Abstract

The chemical profile of *Ajania tibetica* essential oil (EO) and its phytotoxic, insecticidal, and antimicrobial activities were assessed. Monoterpenes (79.05%) and sesquiterpenes (10.33%) were dominant in the EO, with camphor, (+/-)-lavandulol and eucalyptol being the major constituents, representing 55.06% of the total EO. The EO possessed potent phytotoxicity against *Poa annua* and *Medicago sativa* starting from 0.5 mg/mL, and when the concentration rose to 5 mg/mL, seed germination of both tested species was 100% suppressed. *Ajania tibetica* EO displayed significant pesticidal activity against *Aphis gossypii* with an LC_50_ value of 17.41 μg/mL; meanwhile, the EO also showed antimicrobial activity against *Escherichia coli*, *Bacillus subtilis*, *Verticillium dahlia* and *Aspergillus niger* using broth microdilution and disc diffusion methods. For the tested bacterial and fungal strains, the EO exhibited a repressing effect, with minimum inhibitory concentrations (MICs) ranging from 0.3125 to 1.25 mg/mL for bacteria and from 1.25 to 2.5 mg/mL for fungi, whereas the minimum microbicidal concentrations (MMCs) were 5 mg/mL for bacteria and 2.5 mg/mL for fungi. Our study is the first report on the chemical profile as well as the phytotoxicity, insecticidal and antimicrobic activity of *A. tibetica* EO, indicating its potential value as an alternative synthetic pesticide.

## Introduction

Synthetic chemicals are extensively used in productive activities in agriculture worldwide, but their extensive application has resulted in many challenges, such as the evolution of weed or pest resistance, soil or groundwater pollution, and especially harm to human health. Compared with synthesized compounds, natural products can be alternatives due to their rapid biological degradation ability, low-risk evolution of pest or weed resistance, and weak toxicity to living organisms (Isman, 2015; Pavela and Benelli, 2016).

As natural products, essential oils (EOs), which are secondary metabolites synthesized in plants, have been widely used in safeguarding medical and food applications for many hundreds of years (Suteu et al., 2020; Giunti et al., 2021). As complex volatile liquids, EOs are usually obtained by cold pressing, steam distillation, or mechanical processes (Ferhat et al., 2007) and contain a high diversity of terpenoids and derivatives. The yield and constituents of EOs depend on climatic, ecological, and harvesting period effects, species gene, and extraction technology (Burt, 2004; Wissal et al., 2016). Most aromatic plants produce a large quantity of EOs, which can kill pests and sterilize or suppress the growth of weeds (Insawang et al., 2019; Saleh et al., 2020; Aungtikun et al., 2021; Sousa et al., 2021). Previous reports have demonstrated that certain EOs can produce phytotoxic activity against plants, affecting their seed germination as well as root and shoot growth of seedlings (Dutra et al., 2020; Vasconcelos et al., 2022), causing changes of their protective enzymes’ activity and chlorophyll content (Kong et al., 2021; Han et al., 2021); in addition, the cytotoxicity and aneugenic potential of EOs were evidenced by the reduction of the mitotic index and the presence of chromosomal and nuclear alterations (Singh et al., 2020; Valente et al., 2022). Owing to these properties, some EOs, which are extracted from aromatic plants, have the potential value to be further used as environmentally friendly alternatives to synthesized insecticides, weedicides, or bactericides. As a successful commercial example, clove oil was a main active ingredient in the Burnout II herbicide (Bonide Products Inc., Oriskany, NY, USA). Another commercial product, “Rice Weevil Eradication” (producer: Hub Club, Siheung, Korea), contains an active ingredient of cinnamon (*Cinnamomum cassia* Bark) oil (Yang et al., 2020).

*Ajania*, a genus of the Compositae family, comprises approximately 30 species that are perennial herbs or small semishrubs. Most *Ajania* species are aromatic and can be used as folk medicine; moreover, they have been used in dispelling wind and sedation, clearing heat, relieving cough, reducing swelling and bleeding, diminishing inflammation and itching, and repelling mosquitoes and killing insects (Wu et al., 2015; Wangchuk et al., 2016; Shepherd et al., 2018; Wangchuk et al., 2018). The EOs of *Ajania* species, including *A. nubigena* (Wallich ex Candolle) C. Shih, *A. fruticulosa* (Ledeb.) Poljak., *A. przewalskii* Poljak., and *A. semnanensis* Sonboli, have been confirmed to possess antimicrobial activities (Wangchuk et al., 2013; Salehi et al., 2015; Sampietro et al., 2017); moreover, it have been reported that the EOs of *Ajania* species also display other activities, including insecticidal and antagonistic activity (from *A. nitida* Shih, *A. nematoloba* (Hand.-Mazz.) Ling et Shih, *A. potaninii* (Krasch.) Poljak. and *A. fruticulosa*) (Li et al., 2018; Liang et al., 2018; Shao et al., 2021).

*Ajania tibetica* (Hook.f. & Thomson) Tzvelev is distributed in the Tibet, Sichuan and Xinjiang provinces of China, growing at an elevation of approximately 3000-5000 m. It has also been discovered in India and some countries of Central Asia, such as Pakistan, Tajikistan, Kazakhstan, Afghanistan, Kyrgyzstan, and the Syrian Arab Republic (https://www.cvh.ac.cn; https://www.gbif.org). It was reported that *A. tibetica* was a controversial taxonomic status and should be merged into *Phaeostigma* resulting from the pollen morphology of representative species of *Phaeostigma* and *Ajania* genera (Huang et al., 2017). Some reports have shown that *A. tibetica* and *Ceratoides compacta* (Losinsk.) Tsien et C. G. Ma are the main species forming the alpine desert vegetation in the Qaidam Basin, which lives at 2600~3200 m in altitude (Wu et al., 2011) and is also frequently found in sandy gravelly deserts (Hou, 1983). Although there have been some reports regarding the research of *A. tibetica* species as above, very little is known about the physiological and biochemical characteristics of *A. tibetica*.

To date, the chemical compositions and bioactivity characteristics of *A. tibetica* EO are unknown. The purposes of our study were as follows: (i) to evaluate the chemical profile of *A. tibetica* EO and (ii) to determine the phytotoxicity and insecticidal and antimicrobial activity of *A. tibetica* EO. It is expected to lay a foundation for utilizing *A. tibetica* EO as a botanical herbicide, insecticide or fungicide.

## Materials and methods

### Experimental material

The aerial parts of *A. tibetica* at the flowering stage were sampled from Taxkorgan County in southeastern Pamir Plateau, Xinjiang Province, China, in August 2021. Plant samples were air-dried in the laboratory before they were used. It was identified by Dr. Cao Qiumei from Xinjiang Institute of Ecology and Geography, Chinese Academy Sciences, and a voucher specimen was deposited at the herbarium of Xinjiang Institute of Ecology and Geography, Chinese Academy of Sciences.

Seeds of *Medicago sativa* L. and *Poa annua* L. were collected in suburban Urumqi city in 2020 and were surface sterilized using 75% alcohol for 3 min, followed by rinsing with distilled water 3 times before the phytotoxic activity assay (Han et al., 2021)

*Aphis gossypii* Glover was collected from *Solanum nigrum* L. plants and used as a tested pest prepared for the detection of insecticidal activity of *A. tibetica* EO.

The antimicrobial activity of *A. tibetica* EO against bacteria (*Bacillus subtilis* (Ehrenberg) Cohn CICC 21897, *Escherichia coli* (Migula) Castellani et Chalmers CICC 10305) and fungi (*Aspergillus niger* van Tiegh CICC 41255, *Verticillium dahlia* Kleb. V991) was measured. The *V. dahlia* strain was isolated from the rhizosphere soil of cotton plants growing in Urumqi, Xinjiang, and identified by Dr. Yang Honglan according to its morphological characteristics combined with molecular identification, and kept at Xinjiang Institute of Ecology and Geography, Chinese Academy Sciences, China. Other strains were purchased from the China Center of Industrial Culture Collection, CICC (http://m.China-cicc.org).

### EO extraction

*Ajania tibetica* EO was obtained using the steam distillation method with a Clevenger apparatus for 4 h. The extracted EO was stored in a brown vial at 4°C for further study. The *A. tibetica* EO production was determined by the following formula:


$$\text{Oil yield}\left(\%,\frac{V}{W}\right)=\frac{\text{volume of EOs (mL)}}{\text{dried weight of plants (g)}}\times 100\%$$

The constituents of the EO extracted from *A. tibetica* plants were detected by a 7890A/5975C gas chromatography−mass spectrometry (GC/MS) system (Agilent Technologies, Palo Alto, CA, USA) equipped with an HP-5MS (5%-phenyl)-methylpolysiloxane phase column (30 m×0.25 mm; film thickness 0.25 μm) with helium, a carrier gas, with a flow rate of 1 mL/min. The oven temperature was initiated at 50°C for 10 min then programmed from 50°C to 120°C at a rate of 1.5°C/min; from 120°C to 240°C at a rate of 20°C/min and then maintained at this temperature for 5 min. Mass spectra were taken at 70 eV. Mass range was m/z 35–600 Da. The temperature of both detector and injector were held at 280°C. The compounds were determined by comparing their mass spectra and retention indices (RIs) with the data stored in the NIST database (National Institute of Standards and Technology). The retention index was calculated using linear interpolation relative to retention times of a standard mixture of C_7_–C_40_ n-alkanes, as following formula: RI=100[n + (N - n) x (Log RT (unknown) – log RT (n))/log RT(N) – log RT (n)], was used; where n=no. of carbon atoms in the smaller alkane, N=no. of carbon atoms of the larger alkane, RT=retention time of the individual compound (Han et al., 2021; Zhou et al., 2021).

### Phytotoxic activity

*Poa annua* and *M. sativa* were used to examine the phytotoxic effect of the EO. *Ajania tibetica* EO was dissolved in Tween 20 (final concentration 0.1%) to obtain solutions at 0.25, 0.5, 1, 2, and 5 mg/mL for the bioassay. Three milliliters of solution were spread onto each Petri dish (9 cm in diameter), and distilled water containing 0.1% Tween-20 was used as the control, followed by sowing 20 seeds of the test weeds. Petri dishes were placed in a growth incubator at 25°C with a 16 h/8 h light/dark photoperiod for 7 days. The seedlings of *M. sativa* and *P. annua* were measured after 7 days. There were 3 repetitions for the assay, and a total of 60 seedlings were measured for each treatment.

### Insecticidal Activity

The insecticidal activity of *A. tibetica* EO was assessed according to Zhou et al. (2021)’s method with minor modifications. *Ajania tibetica* EO was dissolved in 0.1% Tween-20 solution to achieve concentrations of 5, 10, 20, 40, 80, and 100 μg/mL suspension, which were then impregnated into the paper discs (Whatman #2, USA, 1 × 1 cm). The paper discs were then tapped into the inner side of each Petri dish lid (9 cm in diameter) to separate the EO from *A. gossypii*. Thirty adults of *A. gossypii* were placed on a fresh healthy leaf of *S. nigrum* plants on a layer of wet filter paper. Petri dishes were sealed using Parafilm^®^ film and placed in a growth incubator set at 25°C temperature and a photoperiod of 16 h/8 h light/dark for 2 days. The lethal rate of *A. gossypii* adults was tested at 24 h intervals after treatment. Each treatment was performed in triplicate.

### Antimicrobial activity

#### Diffusion method

The inhibitory effect of *A. tibetica* EO was evaluated using the disc diffusion method according to Lu et al. (2018) with minor modifications. All bacteria were cultured in Luria-Bertani (LB) agar medium at 37°C for 24 h, while fungi were cultured in potato dextrose agar (PDA) at 28°C for 7 days to obtain the fungal spore solution. The active bacteria were prepared in LB broth to obtain 1× 10^8^ colony forming units/mL; the active fungal spores were also cultivated on potato dextrose broth (PDB) to obtain 1× 10^8^ colony forming units/mL using the cell counting in the blood ball counting board; then, one hundred microliters of bacterial/fungal broth were spread on the surface of the agar plates prepared previously. One milliliter of *A. tibetica* EO solutions with concentrations of 5, 10, 20 and 40 µg/mL prepared in 0.1% Tween-20 was impregnated on serialized 5 mm diameter paper discs (Whatman#2, USA), which were then placed in agar plates (the controls received 0.1% Tween-20 solution) and incubated at 37°C for 24 h for bacteria and 28°C for 48 h for fungi. The diameter of the zone inhibition was measured. Each treatment was conducted in triplicate.

#### Determination of MIC and MMC

The minimum inhibitory concentration (MIC) and the minimal microbicidal concentration (MMC) were confirmed using Teh et al. (2017)’s method with some modifications. The EO was prepared in 0.1% Tween-20 to yield the following concentrations: 0.3125, 0.625, 1.25, 2.5, 5, and 10 mg/mL. The reaction was achieved by mixing 100 µL of different concentration solutions with 100 µL of microbial suspension on a 96-well microtiter plate; the controls received 200 µL of 0.1% Tween-20 solutions. The fungal and bacterial plates were then incubated at 28°C for 48 h and 37°C for 24 h, respectively. The optical density (OD) value of the mixed solution was measured at an absorbance of 600 nm by a multimode microplate reader (Varioskan^®^ Flash, Thermo Fisher Scientific Technology Co., Ltd, China). The MIC was detected by considering the OD values of the mixed solutions compared with those of the controls. Meanwhile, MMC was also confirmed by a mixed solution from the well with relatively low OD values and spreading it on LB or PDA plates to incubate at 37°C for 24 h for bacteria and 28°C for 48 h for fungi, thereby determined by assessing the plates without microbial colonies. Three replicates were prepared for each treatment.

### Statistical analysis

One-way ANOVA (*P<* 0.05) was applied to measure the phytotoxic, insecticidal and antimicrobial activities of the EO at different concentrations using SPSS statistics software (IBM SPSS Statistics for Windows, Version 23.0). PROBIT analysis was used to calculate moderate inhibition/lethal (IC_50_) values for the inhibitory/lethal concentration (SAS/STAT User’s Guide; SAS Institute Inc., Cary, NC, USA).

## Results

### Yield and Composition of *A. tibetica* EO

*Ajania tibetica* EO was extracted from the dry aboveground plant materials by the traditional steam distillation method. The yield was 0.01% (v/w, volume/dry weight). Eventually, forty-nine constituents were confirmed, which shared 95.93% of the total oil, whereas 4.07% of the oil remained unclassified (**Table 1**). The major compounds were camphor (29.76%), (+/-)-lavandulol (13.23%), and eucalyptol (12.07%), which represented 55.06% of the total oil. In general, the EO was composed of 79.05% monoterpenes (including 11.41% monoterpene hydrocarbons and 67.64% oxygenated monoterpenes) and 10.33% sesquiterpenes (including 0.18% sesquiterpene hydrocarbons and 10.15% oxygenated sesquiterpenes) (**Table 1**).

**Table 1:** The chemical profile of *A. tibetica* EO.

Peaks	RT	Compound name	RI ^a^	RI ^b^	Area(%)
1	4.61	Isobutyl isobutyrate	914	913	0.04
2	4.86	(1*S*)-(+)-3-Carene	928	929	0.15
3	4.99	(1*S*)-(-)-alpha-Pinene	936	937	2.11
4	5.25	Camphene	952	952	1.88
5	5.64	Sabinene	977	976	1.37
6	5.71	β-Pinene	978	981	0.45
7	5.81	5-Hepten-2-one, 6-methyl-	988	987	0.28
8	5.89	β-Myrcene	991	992	0.18
9	6.09	Butanoic acid, 2-methyl-, 2-methylpropyl ester	1004	1003	0.05
10	6.16	α-Phellandrene	1007	1008	0.63
11	6.31	Propanoic acid, 2-methyl-, 2-methylbutyl ester	1017	1016	0.16
12	6.38	2-Carene	1011	1020	0.88
13	6.52	o-Cymene	1028	1028	1.15
14	6.60	Limonene	1030	1032	0.30
15	6.66	Eucalyptol	1033	1035	12.07
16	7.12	γ-Terpinolene	1061	1062	1.35
17	7.28	(1alpha,2alpha,5alpha)-2-methyl-5-(1-methylethyl)bicyclo[3.1.0]hexan-2-ol	1071	1071	0.46
18	7.66	Terpinolene	1092	1092	0.97
19	7.84	(1alpha,2beta,5alpha)-2-methyl-5-(1-methylethyl)bicyclo[3.1.0]hexan-2-ol	1099	1102	0.51
20	7.89	2-Methylbutyl 2-methylbutyrate	1102	1105	0.25
21	8.02	4-Methyl-1-pentylisobutyrate	1110	1112	0.05
22	8.27	cis-4-(isopropyl)-1-methylcyclohex-2-en-1-ol	1124	1126	0.45
23	8.35	Campholenic aldehyde	1131	1131	0.12
24	8.44	4-Acetyl-1-methylcyclohexene	1131	1135	0.15
25	8.61	2-Cyclohexen-1-ol, 1-methyl-4-(1-methylethyl)-, cis-	1139	1145	0.40
26	8.75	Camphor	1153	1153	29.76
27	8.80	Bicyclo[2.2.1]heptan-2-ol, 2,3,3-trimethyl-	1150	1156	0.09
28	8.86	Cyclohexanone,5-methyl-2-(1-methylethyl)-, (2R,5S)-rel-	1159	1159	0.12
29	9.06	(+/-)-Lavandulol	1170	1170	13.23
30	9.10	Borneol	1172	1173	1.53
31	9.30	3-Cyclohexen-1-ol,4-methyl-1-(1-methylethyl)-, (1R)-	1175	1183	5.70
32	9.41	2-(3-methylphenyl)propan-2-ol	1186	1190	0.37
33	9.52	α-Terpineol	1195	1196	1.67
34	9.60	2-Cyclohexen-1-ol, 3-methyl-6-(1-methylethyl)-, cis-	1203	1201	0.25
35	9.81	2-Cyclohexen-1-ol, 3-methyl-6-(1-methylethyl)-, trans-	1213	1212	0.16
36	10.01	2-Cyclohexen-1-ol, 2-methyl-5-(1-methylethenyl)-, cis-	1222	1224	0.14
37	10.38	Pulegone	1245	1246	0.23
38	10.45	D-Carvone	1249	1250	0.08
39	11.17	lavandulyl acetate	1292	1291	4.00
40	12.00	cis-2-methyl-5-(1-methylvinyl)cyclohex-2-en-1-yl	1381	1341	0.10
41	13.08	Methyleugenol	1406	1406	0.09
42	13.35	Lavandulyl isobutyrate	1417	1424	1.21
43	13.47	Caryophyllene	1428	1431	0.18
44	15.55	Nerolidol	1566	1567	0.09
45	15.91	Spathulenol	1582	1591	0.23
46	16.00	Caryophyllene oxide	1592	1598	0.47
47	16.97	β-Eudesmol	1662	1666	6.93
48	17.34	α-Bisabolol	1696	1693	2.43
49	18.08	Chamazulene	1742	1747	0.48
		Monoterpene hydrocarbons			11.41
		Oxygenated monoterpenes			67.64
		Sesquiterpene hydrocarbons			0.18
		Oxygenated sesquiterpenes			10.15
		Others			6.56
		Total identified			95.93
		Oil yield (%, V/W)			0.01

RT, Retention time. RI ^a^: Retention index calculated by linear interpolation relative to retention times of a standard mixture of n-alkanes (C_7_–C_40_) using a HP-5MS column. RI ^b^: Retention index from literature. Area (%): Percentage of the constituents.

### Phytotoxicity Bioassay

**Figure 1:**
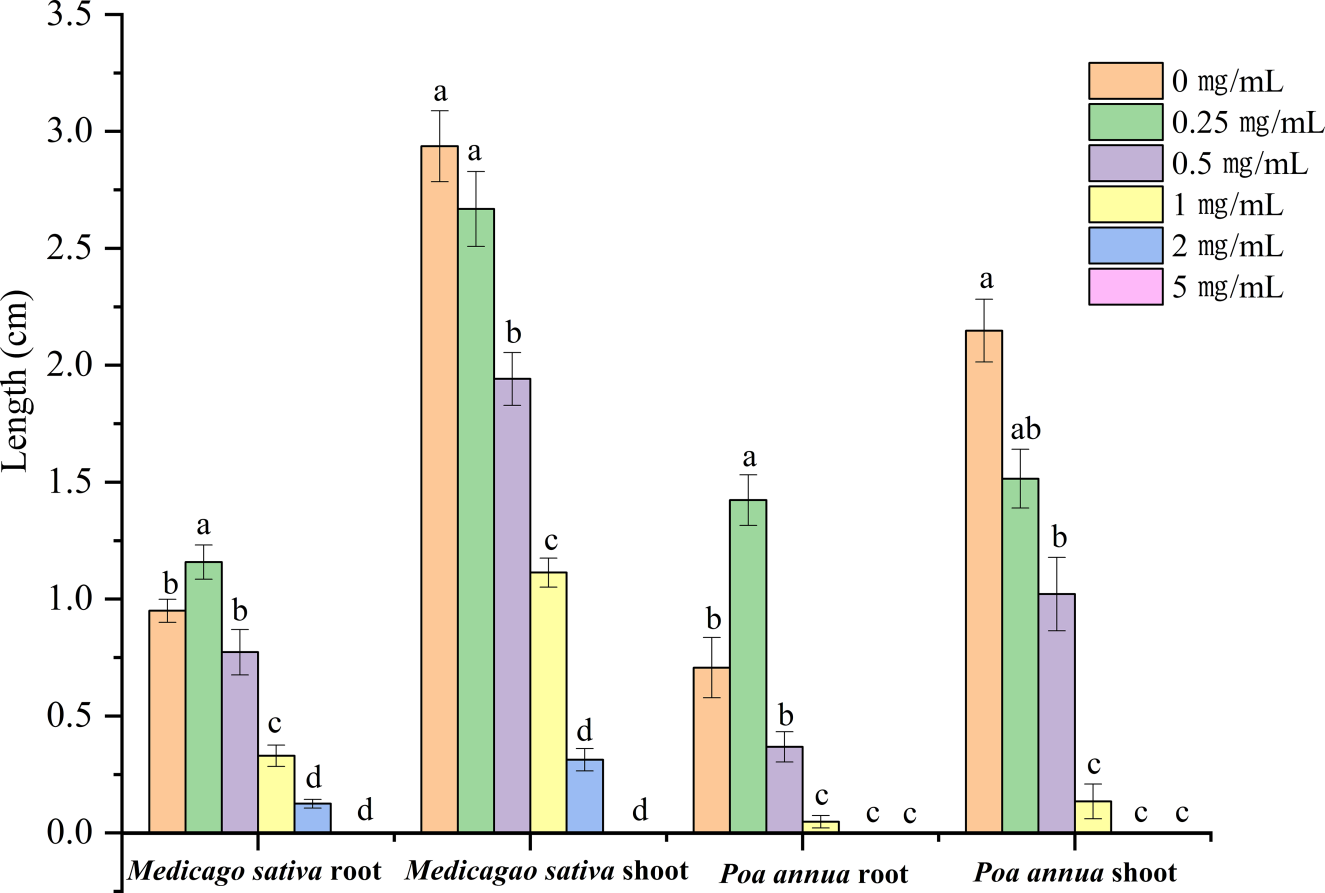
Phytotoxic effects of *A. tibetica* EO on seedling growth of *P. annua* and *M. sativa* (n = 60). Different letters represent a significant difference at *P*< 0.05 level according to Fisher’s LSD test.

**Figure 2:**
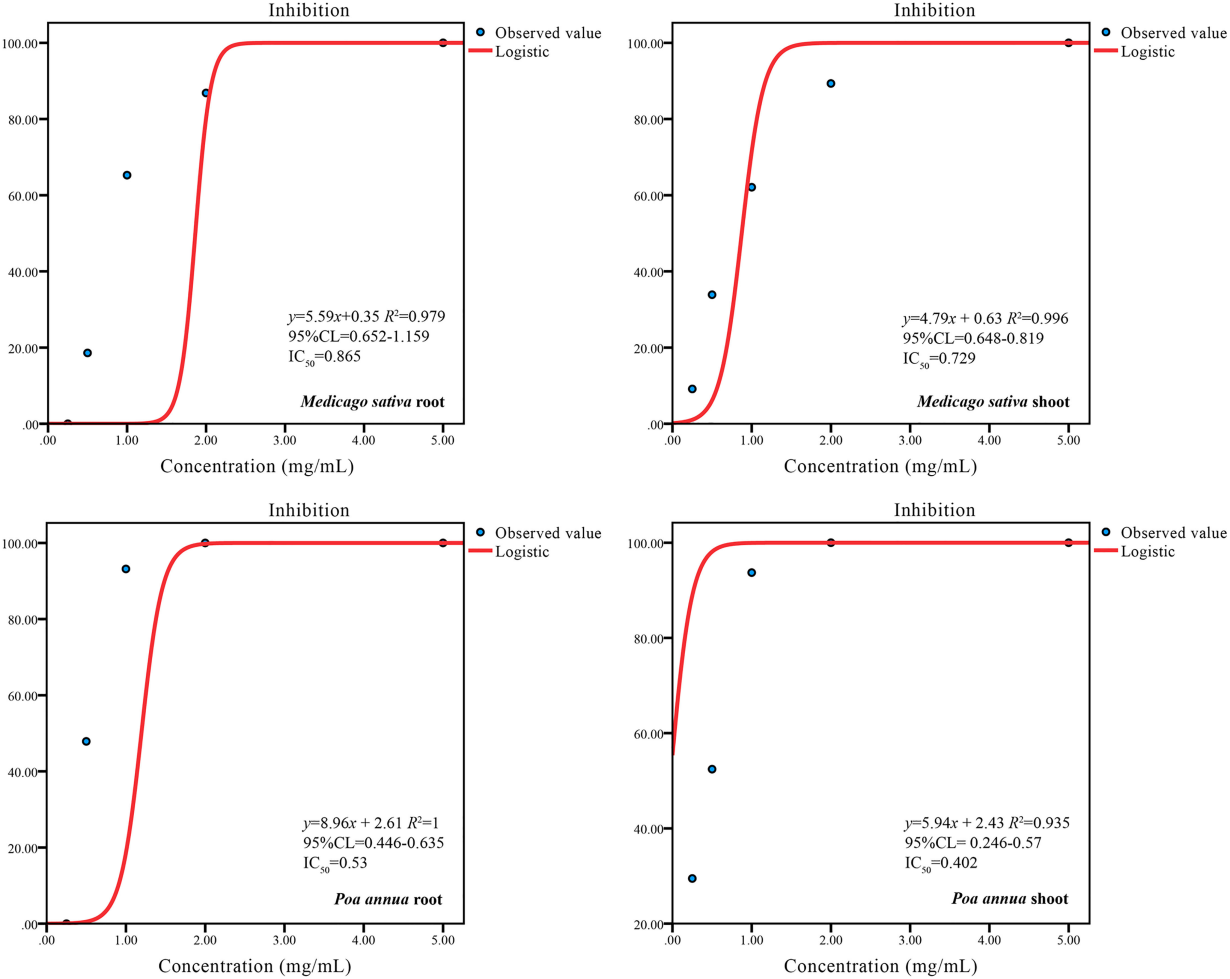
Dose–response curves of *A. tibetica* EO on seedling growth of *M. sativa* and *P. annua*. *R^2^* adj: adjusted coefficient of determination. IC_50_: 50% inhibit concentration of bested plants. 95% CL: 95% confidence limits.

The phytotoxicity of the EO (concentrations ranged from 0 to 5 mg/mL) was estimated by comparing their plant growth regulating effect on *M. sativa* and *P. annua*. In general, the EO impacted the growth of the tested plants in a dose-dependent manner. Root development of *M. sativa* and *P. annua* was promoted at a concentration of 0.25 mg/mL and then reduced with increasing EO concentration, although the EO started to inhibit shoot growth of *M. sativa* and *P. annua* at 0.25 mg/mL. Under the treatment with 0.5 mg/mL, significant inhibition of root and shoot development was observed in both test species. Meanwhile, the seed germination of both tested plants was completely suppressed at the highest concentration of 5 mg/mL tested (**Figure 1**). In detail, *M. sativa* and *P. annua* root length were promoted by 37.89% and 101.40% at the concentration of 0.25 mg/mL EO, respectively. When the treatment with 0.5, 1, 2, and 5 mg/mL EO was compared to the control, the root length of *M. sativa* significantly declined by 7.94%, 60.71%, 85.12%, and 100%, respectively, and the root length of *P. annua* decreased by 47.88%, 93.17%, 100%, and 100%, respectively (**Figure 1**). Similarly, *M. sativa* shoot length was significantly inhibited by 9.14%, 33.88%, 62.09%, 89.33% and 100%, and *P. annua* shoot length was suppressed by 29.48%, 52.44%, 93.72%, 100%, and 100% when treated with 0.25, 0.5, 1, 2, and 5 mg/mL EO in comparison to the control. The EO showed an inhibitory effect with IC_50_ values of 0.865, 0.729, 0.53, and 0.402 mg/mL on the root and shoot growth of *M. sativa* and *P. annua*, respectively (**Figures 1**, **2**). The dose–response curve of the phytotoxic activity is shown in **Figure 2**.

### Pesticidal Activity

**Figure 3:**
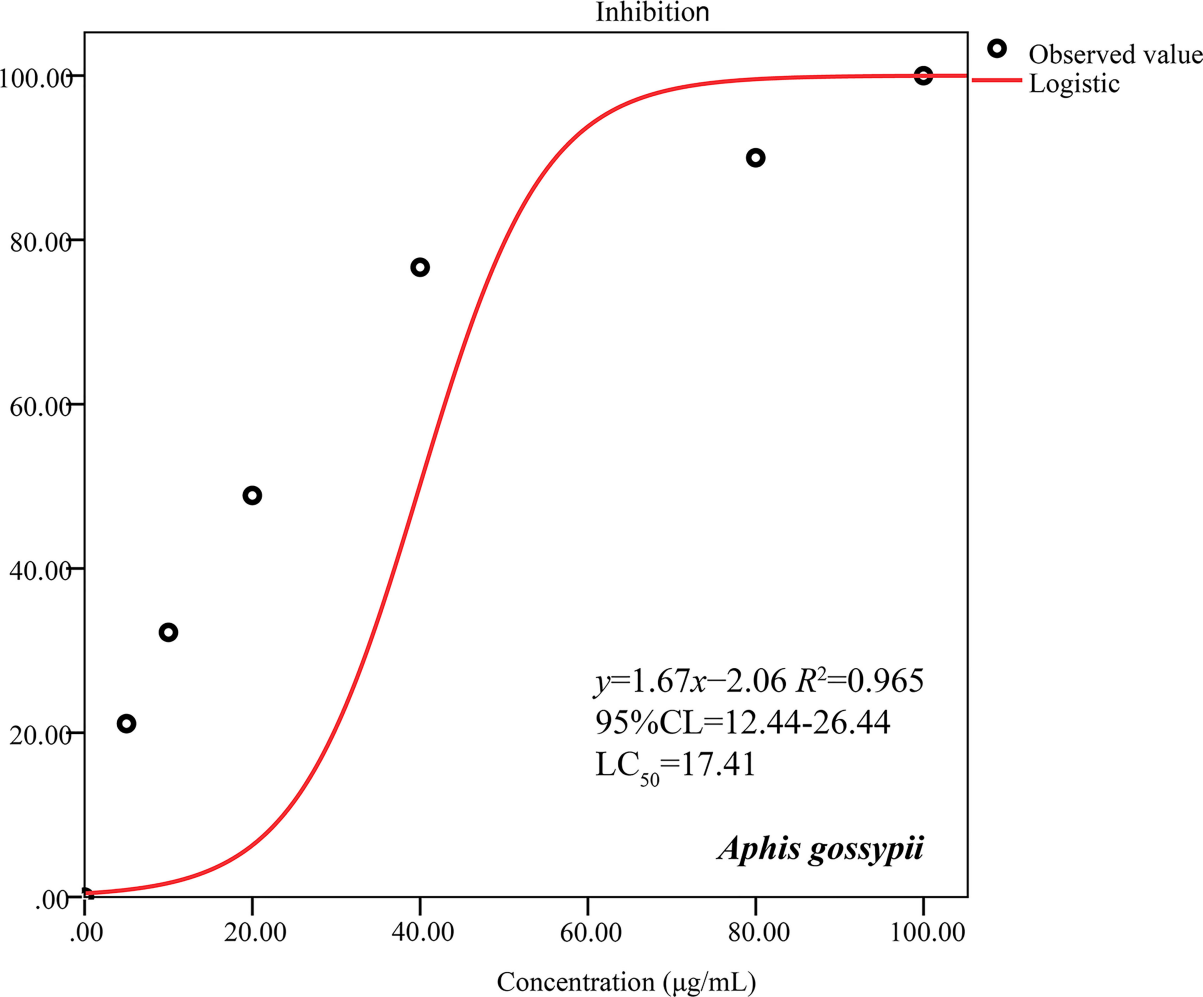
Dose–response curves of *A. tibetica* EO against *A. gossypii* adults. *R^2^* adj: adjusted coefficient of determination. LC_50_: 50% lethal concentration of *A. gossypii*. 95% CL: 95% confidence limits.

The pesticidal activity of *A. tibetica* EO was assessed on adjusted mortality rates of *A. gossypii* at concentrations ranging from 0 to 100 μg/mL. The results showed that the EO exerted lethal effects and induced obvious behavioral avoidance in *A. gossypii*. The EO completely killed all the tested insects at a dose of 100 μg/mL after 24 h of exposure. The mortality rates of *A. tibetica* EO reached 21.11%, 32.22%, 48.89%, 76.67%, and 90.00%, respectively, under 5, 10, 20, 40, and 80 μg/mL EO treatments for 24 h of application. The EO exhibited significant pesticidal activity against *A. gossypii* with an LC_50_ value of 17.41 μg/mL (**Figure 3**). The dose–response curve of the insecticidal activity is shown in **Figure 3**.

### Antimicrobial activity

**Figure 4:**
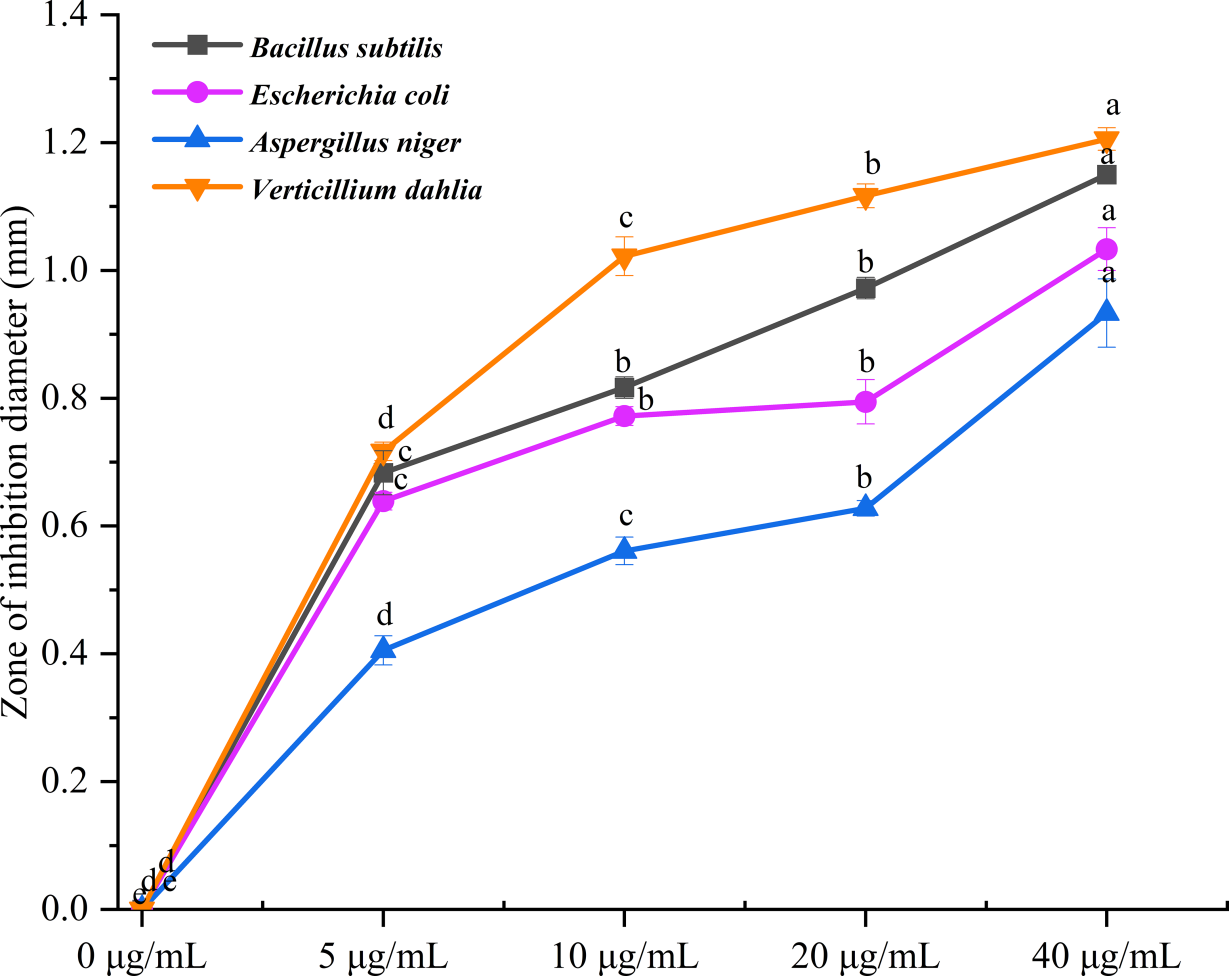
Antimicrobial activity of *A. tibetica* EO. Different letters indicated significant differences (*P<* 0.05) level according to Fisher’s LSD test.

**Figure 5:**
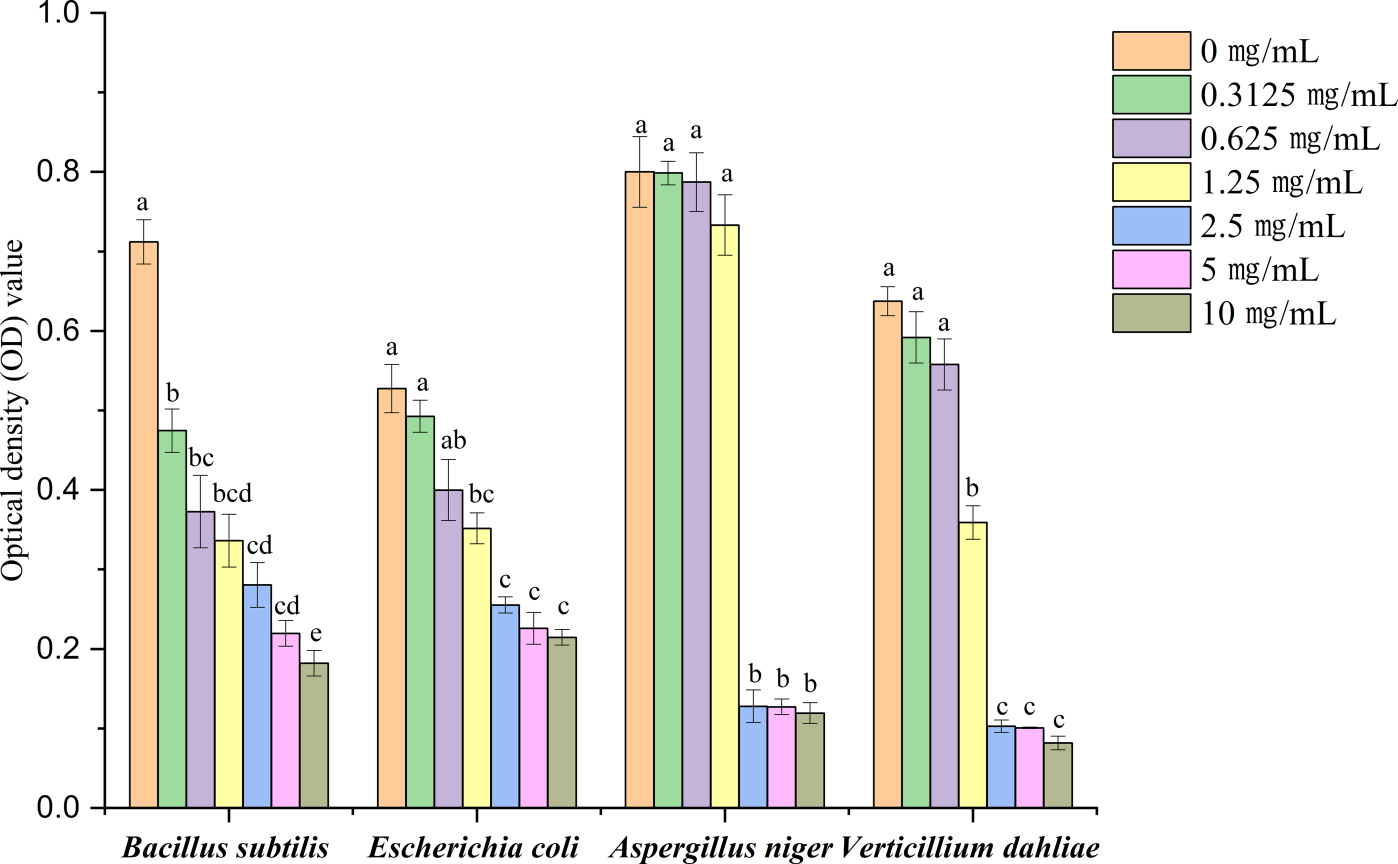
The optical density (OD) value on antimicrobial activity of *A. tibetica* EO. Different letters indicated significant differences (*P*< 0.05) level according to Fisher’s LSD test.

**Figure 6:**
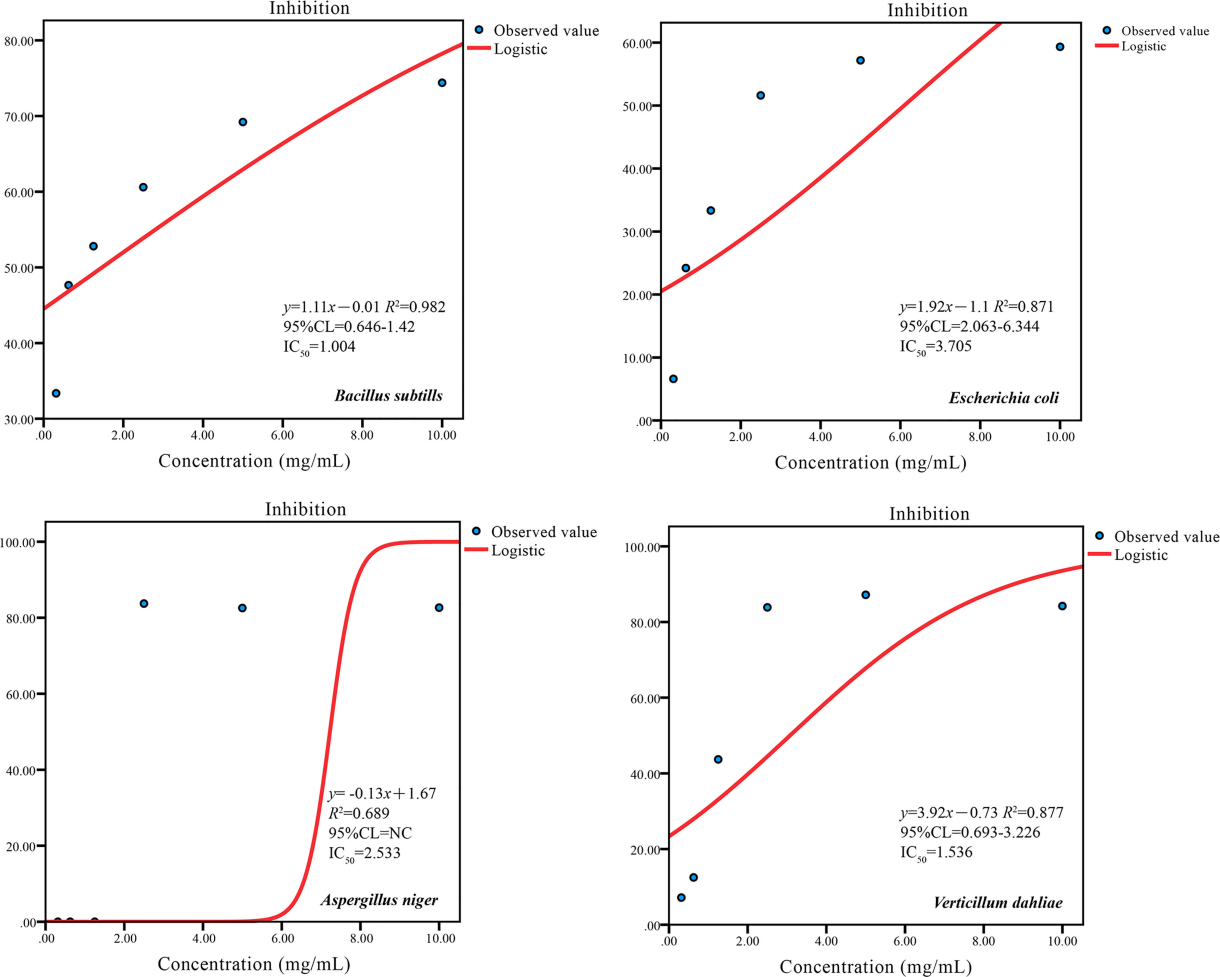
Dose–response curves of *A. tibetica* EO against tested microorganisms. *R^2^* adj: adjusted coefficient of determination. IC_50_: 50% inhibit concentration of microorganisms. 95% CL: 95% confidence limits; NC, not calculable.

The antimicrobial activity of *A. tibetica* EO against 4 microorganisms was estimated using both the disc diffusion and broth microdilution methods. All the tested microorganisms were suppressed by the EO, as the diameter of the zone of inhibition significantly increased with increasing EO concentration. The results from the disc diffusion method indicated that *B. subtilis* of the bacterial strains was the most sensitive to the high concentration of 40 µg/mL of *A. tibetica* EO with a zone diameter of 1.15 mm compared to those obtained from *E. coli* with 1.03 mm; in addition, *V. dahliae* with a zone diameter of 1.21 mm in the fungal strains was more sensitive than *A. niger* with 0.93 mm at the high concentration of 40 µg/mL of *A. tibetica* EO (**Figure 4**). In addition, the optical density (OD) values of antimicrobial activity of *A. tibetica* EO against all the test microorganisms declined with increasing concentration, which also showed an inhibitory effect of the EO on the tested microorganisms. The optical density (OD) values of all the tested microorganisms were significantly reduced at a concentration of 2.5 mg/mL EO (**Figure 5**). The IC_50_ values of the EO inhibited *B. subtilis*, *E. coli*, *A. niger*, and *V. dahliae* were 1.004, 3.705, 2.533, and 1.536 mg/mL, respectively (**Figure 6**). The dose–response curve of the antimicrobial activity is shown in **Figure 6**. The MICs for *B. subtilis*, *E. coli*, *A. niger*, and *V. dahliae* were 0.3125, 1.25, 2.5, and 1.25 mg/mL, respectively; the MIC of the bacteria ranged from 0.3125 to 1.25 mg/mL, whereas the MIC for fungi ranged from 1.25 to 2.5 mg/mL. The results of the MMC test indicated that *A. tibetica* EO had MMC values of 2.5 and 5 mg/mL for all tested bacteria and fungi, respectively.

## Discussion

The results on the chemical profile of EO obtained from *A. tibetica* were distinct from other species of the *Ajania* genus in previous reports. Previously, 1,8-cineole and camphor were determined to be the main chemical constituents in no less than 5 species of *Ajania* plants (Shatar et al., 2010; Salehi et al., 2015; Shao et al., 2021). The EO of *A. nematoloba* revealed beta-pinene (34.72%), eucalyptol (24.97%), and verbenol (20.39%) as the major compounds, whereas the main components of *A. nitida* EO were camphor (20.76%), thujone (18.64%), eucalyptol (13.42%) and borneol (8.32%); there were differences in terms of type and amount of main component in the EOs of *A. nematoloba* and *A. nitida* (Li et al., 2018). In addition, myrcene (19.1%), 1,8-cineole (34.2%), and –pinene (9.4%) were found to be the main compounds in *A. fruticulosa* EO reported by Sampietro et al. (2017), while Liang et al. (2016) previously revealed that the main constituents of *A. fruticulosa* EO were myrtenol (8.15%), (+)-camphor (32.10%), and 1,8-cineole (41.40%). Hence, it was demonstrated that there were also differences in the principal components of essential oils extracted from the same *A. fruticulosa* species.

Similarly, in the present work, the chemical composition of *A. tibetica* EO was different from other *Ajania* species. For instance, the main constituents of *A. tibetica* EO were camphor, (+/-)-lavandulol, and eucalyptol, compared with *A. nitida* EO whose major constituents were camphor, thujone, eucalyptol and borneol; the relative percentage of eucalyptol in *A. tibetica* EO (12.07%) was less than that in *A. nitida* EO (13.42%) and *A. nematoloba* EO (24.97%), respectively, whereas camphor was the most abundant component in *A. tibetica* EO (29.76%), compared with 20.76% camphor in *A. nitida* EO reported by Li et al. (2018). Moreover, previous studies have demonstrated the diversity of essential oil profiles of *Ajania* plants; for example, Liang et al. (2016) described that the content of 1,8-cineole was 41.4% in *A. fruticulosa* EO growing in China, while it was 34.2% in those cultivated in Kazakhstan by Sampietro et al. (2017). On the other hand, Salehi et al. (2015) found that the chemical composition of *A. semnanensis* EO varied with the different growth stages. These results revealed that species belonging to the same genus usually have specific volatile components. Meanwhile, a number of biotic and abiotic factors including growing stages, geography, light, temperature, water, nutrient conditions, climatic conditions, etc. might also affect the EOs’ chemical profiles, thereby leading to differences in the biosynthetic pathways of the plant, chemotypes, compounds, and contents (Zheng et al., 1999; Han et al., 2021).

It has been reported that plant-derived EOs and their constituents possess phytotoxic activity against seed germination and seedling growth of tested species (Langenheim, 1994; Vokou et al., 2003; Salamci et al., 2007; Abd-Elgawad et al., 2021). However, to the best of our knowledge, the phytotoxic activity of EOs obtained from *Ajania* plants have not yet been evaluated. In the present work, we found *A. tibetica* EO exhibited inhibitory effects on the tested species in a dose-dependent manner. Under 0.5 and 1 mg/mL *A. tibetica* EO treatment, the root length of *P. annua* decreased by 47.88% and 93.17%, respectively. In comparison, glyphosate as a commercial herbicide presented stronger phytotoxic effect, inhibiting *P. annua*’s root elongation by 79.08% at 0.25 mg/mL and 93.44% at 0.5 mg/mL (Wei et al., 2020). For the dicot plant *M. sativa*, *A. tibetica* EO suppressed its root length by 7.94%, 60.71%, 85.12%, and 100% under 0.5, 1, 2, and 5 mg/mL treatment respectively, which was similar to the inhibitory effect of *Artemisia absinthium* EO and *Ambrosia artemisiifolia* EO on root growth of *M. sativa* in previous reports by Jiang et al. (2021) and Han et al. (2021); however, at the dose of 0.25mg/mL, *A. tibetica* EO promoted *M. sativa* root length by 37.89%, which were completely different from the suppressing effect of *A. absinthium* EO and *A. artemisiifolia* EO. These results revealed that *A. tibetica* EO exhibited different biological activity compared with other species. Additionally, the phytotoxic activity of *A. tibetica* EO could be ascribed to the diversity of chemical constituents in *A. tibetica* EO, especially the monoterpene compounds (79.05%) compared with sesquiterpenes (10.33%), which were the main class of terpenoids in *A. tibetica* EO. Some earlier studies reported that monoterpenes, including monoterpene hydrocarbons and oxygenated monoterpenes, suppressed the growth of many crops and weeds (Lopez et al., 2008; Li et al., 2011; Ali et al., 2015); it was also reported that the phytotoxic activity of oxygenated monoterpenes could be much stronger than that of monoterpene hydrocarbons (Kordali et al., 2007; Amri et al., 2017). Moreover, it was confirmed that the monoterpenes camphor (29.76%) and eucalyptol (12.07%), the main compounds of *A. tibetica* EO, exhibited phytotoxic activity against the tested plants in previous reports (Shao et al., 2018). It has also been reported that sesquiterpene compounds possessed strong phytotoxic potential; for example, roots treated with farnesene was negatively affected with obvious tissue and cellular alterations and morphological modifications (Araniti et al., 2016). Therefore, it needs to be further confirmed whether the observed phytotoxicity of *A. tibetica* EO is attributed to the monoterpene compounds in the EO.

Phytochemicals play pivotal roles in pest management action for agricultural sustainability (Duke et al., 2003; Boulogne et al., 2012; Gahukar, 2014). Previously, EOs obtained from *Ajania* species were confirmed to possess pesticidal activity, such as, *A. nematoloba* and *A. nitida* EO showed contact toxicity with LD_50_ values of 102.29 and 30.10 µg/adult, respectively, and fumigant toxicity with LC_50_ values of 69.45 and 20.07 mg/L, respectively, against the red flour beetle *Tribolium castaneum* Herbst after 24 h of exposure (Li et al., 2018). *Ajania potaninii* EO was also evaluated for pesticidal activity against *Plodia interpunctella* Hubner, which is a major pest of many economically storage crops (Shao et al., 2021). Our study also found that *A. tibetica* EO exhibited significant pesticidal activity against *A. gossypii* with an LC_50_ value of 17.41 μg/mL for 24 h of application. In addition, *A. fruticulosa* EO also exposed contact effects with LD_50_ values of 89.85 g/cm^2^ and 105.67 g/adult and fumigant effects with LC_50_ values of 0.65 and 11.52 mg/L on *Liposcelis bostrychophila* Badonnel and *T. castaneum* adults for 24 h exposure; moreover, (+)-camphor of its most common compounds exhibited a strong fumigant effect with an LC_50_ of 0.43 mg/L on *L. bostrychophila* (Liang et al., 2016). Similarly, eucalyptol as a monoterpene compound with insecticidal activity, was detected to have a significant contact effect with an LD_50_ of 76.97 μL/mL on the larvae of *Plutella xylostella* L. after 24 h of exposure and strong fumigant activity with an LC_50_ of 3.25 μL/mL against *P. xylostella* adults (Gharib et al., 2020; Huang et al., 2021). Hence, future works is necessary to evaluate whether camphor and eucalyptol are the major components playing a critical role in the insecticidal activity of the EO. Meanwhile, previous studies found that *A. gossypii* was susceptible to diverse EOs. For instance, *Santalum austrocaledonicum* Vieill. EO showed insecticidal activity of 94.0% mortality against *A. gossypii* infesting hot peppers (Roh et al., 2015). It has also been found that EOs of *Pistacia lentiscus* L. and *Mentha pulegium* L. exhibited insecticidal activities against *A. gossypii*, resulting in 70% and 94% mortality rates and LC_50_ values of 759 and 478 ppm, respectively; there was no difference from the toxic effect of the chemical insecticide imidacloprid used as the positive control against *A. gossypii* (Behi et al., 2019), By comparison, *A. tibetica* EO exerted stronger insecticidal activity than imidacloprid. However, it has been found that *Melaleuca styphelioides* Smith EO showed strong fumigant toxicity of 100% mortality against *A. gossypii* adults and nymphs at a concentration of 263.18 μL/L air EO (Albouchi et al., 2018); these results illustrated that *A. tibetica* EO showed much weaker activity than *M. styphelioides* EO. Therefore, future work should focus on comparing the strength of the insecticidal activity of *A. tibetica* EO with commercial pesticides.

The antimicrobial activity of EOs of *Ajania* plants against bacteria and fungi has been previously examined. *Ajania semnanensis* EO exhibited inhibitory effects on bacteria (*E. coli*, *B. subtilis*, *B. cereus*, *Staphylococcus aureus*) and fungi (*Candida albicans*), showing better inhibitory effects of EOs on fungi than bacteria (Salehi et al., 2015). Unlike reported by Salehi et al. (2015), *A.* tibetica EO exhibited antimicrobial activity with the order of *E. coli*>*A. niger*>*V. dahliae*>*B. subtilis* according to IC_50_ values of 3.705, 2.533, 1.536 and 1.004 mg/mL, respectively, which didn’t show an effect pattern on activity on fungi and bacteria. Additionally, *A. nubigena* EO exhibited moderate antifungal activity against *C. albicans* and strong antibacterial activity against *B. subtilis* (compared with the standard, amoxicillin) with minimum inhibition zones of 11 mm and 13 mm, respectively (Wangchuk et al., 2013). Relatively, *A. tibetica* EO exposed much weaker antimicrobial activity against *B. subtilis* with a zone diameter of 1.15 mm than *A. nubigena* EO and amoxicillin. Moreover, *A. fruticulosa* EO also showed an inhibitory effect on strains of *Fusarium verticillioides*, *F. graminearum*, *A. niger* and *A. carbonarius*, while showing no antifungal effects on both *Aspergillus* strains and weak antimicrobial activity against both *Fusarium* strains (Sampietro et al., 2017). These reports demonstrate that EOs from *Ajania* species have different antibacterial activities. *Ajania tibetica* EO showed an inhibitory effect on *B. subtilis*, *E. coli*, *A. niger*, and *V. dahlia* according to the definition of antimicrobic activity of the natural product by Holetz et al. (2002). The antimicrobial activity of *A. tibetica* EO might also be attributed to the synergism of the EO constituents. Therefore, additional studies will be needed to unravel how the components of *A. tibetica* EO play a functional role in antimicrobial activity against the microorganisms.

## Conclusion

The present study demonstrated the phytotoxic, insecticidal and antimicrobial potential of *A. tibetica* EO; in particular, the EO displayed potent suppressive effect on the test weeds and insect, implying that it has the potential to be further explored as eco-friendly agrochemicals for the management of weeds and insects. Future studies should focus on the bioactivity of single/combined constituents of the EO to determine the strength of each compound, and whether synergistic effect occur when different constituents work together; on the other hand, the EO’s phytotoxic effect on other weed species as well as the crops should also be evaluated under field conditions.

## Data availability statement

The original contributions presented in this study are included in the article/supplementary material. Further inquiries can be directed to the corresponding author.

## Author contributions

CH developed the idea for research, with extensive discussion with SZ and YM. CH performed the bioactivity experiments and analyzed its results. SZ, YM and QC collected all experimental material and identified the specimen of *A. tibetica* species plant. KS conducted the analyses relating to antimicrobial activity of *A. tibetica* essential oil. HS edited the manuscript. All authors contributed to the article and approved the submitted version.

## Funding

This research was funded by the National Natural Science Foundation of China (U2003214), the Second Tibetan Plateau Scientific Expedition and Research (STEP) Program(2019QZKK0502), and the Third Xinjiang Scientific Expedition Program (2022xjkk1505).

## Conflict of interest

The authors declare that the research was conducted in the absence of any commercial or financial relationships that could be construed as a potential conflict of interest.

## Publisher’s note

All claims expressed in this article are solely those of the authors and do not necessarily represent those of their affiliated organizations, or those of the publisher, the editors and the reviewers. Any product that may be evaluated in this article, or claim that may be made by its manufacturer, is not guaranteed or endorsed by the publisher.
